# Development of a practical predictive nomogram for cognitive impairment risk following traumatic brain injury: a retrospective cohort study

**DOI:** 10.3389/fnhum.2026.1923498

**Published:** 2026-08-28

**Authors:** Lingling Jiang, Yun Cao, Hao Zhou, Yan Zhang, Jiajia Yin, Lin Li, Xueli Ji

**Affiliations:** Department of Emergency, The First Affiliated Hospital With Nanjing Medical University, Nanjing, China

**Keywords:** cognitive impairment, neuropsychological test battery, prediction model, risk factors, traumatic brain injury

## Abstract

**Background:**

Accurately predicting long-term cognitive impairment following moderate–severe traumatic brain injury (TBI) remains a significant clinical challenge. Existing prognostic tools have limited predictive accuracy for individual-level risk stratification.

**Objective:**

To develop a comprehensive prognostic nomogram for predicting cognitive impairment in adults who suffered a moderate-to-severe TBI.

**Methods:**

We retrospectively included 482 adults with moderate-to-severe TBI admitted to our hospital. All participants completed the standardized 6-month cognitive function assessments at 6 months following TBI using the Neuropsychological Test Battery. Variables were selected from demographic data, clinical factors, laboratory tests and questionnaire scores. A prediction model was developed through multivariable logistic regression analysis.

**Results:**

Cognitive impairment was observed in 28.8% of individuals. The prediction model showed that previous TBI history (OR = 2.002), Marshall CT classification IV (OR = 4.072,)/V (OR = 4.613)/ VI (OR = 5.008), elevated neurological function markers ≥2 (OR = 2.255), elevated proinflammatory cytokines ≥2 (OR = 2.209), Systemic Inflammation Response Index scores (OR = 3.066) and incidence of lower urinary tract symptoms (OR = 4.169), seizures (OR = 5.243) or post-traumatic stress disorders (OR = 5.245) were independent risk factors of cognitive impairment following TBI (all *p* < 0.05). Receiver-operating characteristic curve demonstrated a good discrimination with an area under the curve equaling 0.920. A prognostic index value more than 6.425 were categorized as high-risk to develop cognitive impairment after TBI.

**Conclusion:**

We developed a prognostic nomogram that ‌showed promise for early risk stratification‌ of 6-month cognitive impairment in adults with moderate–severe TBI, utilizing information available during acute hospitalization. ‌While our results were encouraging, external validation in independent, prospective cohorts was required‌ to confirm its generalizability and potential for integration into clinical practice before widespread implementation.

## 1 Introduction

Traumatic brain injury (TBI) refers to an injury to the brain associated with external mechanical forces, including initial impact, rapid acceleration-deceleration or penetration. TBI is typically categories as concussion, contusion, diffuse axonal injury, and intracranial hematoma, involving both closed and penetrating injuries (Ginsburg and Smith, 2026). It is reported that TBI affects nearly 37.93 million individuals in 2021 and remains a leading cause of morbidity and mortality, resulting in a significant health challenge worldwide (Zhong et al., 2025).

The primary injury phase of TBI triggers immediate mechanical damage to brain parenchyma, while secondary pathophysiological cascades including excitotoxicity, ionic disturbances, reduced cerebral blood flow, cerebral oedema, oxidative stress and neuroinflammation persist for months or even years following the initial insult (Orr et al., 2024). As the most common long-term consequence, cognitive impairment spanning domains of attention, arousal, concentration, executive functioning, memory, behavior or mood has been observed in more than two-thirds of survivors of moderate-to-severe TBI, and these persistent deficits substantially disrupt patients’ daily life and social function (Himanen et al., 2006).

Mounting evidence has confirmed that timely cognitive rehabilitation, structured exercise and targeted pharmacological therapies can effectively mitigate post-TBI cognitive deficits, yet the clinical benefit of these interventions is highly dependent on early identification of high-risk populations (Togher et al., 2023). The Repeatable Battery for the Assessment of Neuropsychological Status (RBANS), a brief, widely validated neuropsychological instrument that does not require specialized professional expertise, has been increasingly adopted to quantify global cognitive function across five distinct domains, enabling standardized, objective evaluation of post-TBI cognitive outcomes (Zhang et al., 2022). Previous attempts to build predictive tools for post-TBI cognitive impairment have notable limitations that restrict their real-world clinical utility. A 2025 prospective observational study by Yuan et al., developed a prediction model for post-TBI cognitive impairment, but the nomogram only incorporated basic demographic and routine clinical variables, without integrating multi-dimensional markers spanning demographic characteristics, acute neuroimaging severity grading, peripheral inflammatory markers, neurological injury protein markers, and post-injury clinical complications that are mechanistically linked to long-term neurodegeneration (Yuan et al., 2025).

Given its clinical importance, the validated Chinese version of RBANS was utilized to objectively evaluate 6-month cognitive performance in the included moderate-to-severe TBI cohort. The primary aim of this study was to develop and validate a robust, easy-to-use individualized predictive nomogram that integrates demographic, radiological, laboratory and post-injury complication factors, to accurately stratify patients by their risk of developing cognitive impairment after moderate-to-severe TBI and identify individuals who would derive the greatest clinical benefit from early targeted preventive interventions.

## 2 Materials and methods

The retrospective observational study was approved by the Ethics Committee of The First Affiliated Brain Hospital of Nanjing Medical University (NBH-2026042 on February 16, 2026) in accordance with the Declaration of Helsinki. Written informed consent for data publication was obtained from all patients. Results were reported following the Strengthening the Reporting of Observational Studies in Epidemiology (STROBE) guidelines (Vandenbroucke et al., 2014).

Between January 1, 2023 and July, 312,025, patients admitted for the treatment of TBI were reviewed through the electronic medical records (EMRs). Inclusion criteria were: (1) a confirmed diagnosis of TBI according to the guidelines from Brain Trauma Foundation/American association of Neurological Surgeons (Carney et al., 2017); (2) evidence of intracranial bleeding on computerized tomography (CT) imaging within 24 h after trauma event; (3) a Glasgow Coma Scale (GCS) score of 12 or lower prior to sedation; (4) aged ≥18 years; (5) receiving routine follow-up post-intervention. Patients were excluded if they had non-survival from TBI, uncontrolled bleeding, sustained systolic hypotension <90 mm Hg, sustained tachycardia >120/min, malignancy, severe hepatic/renal dysfunction, primary psychiatric or psychological disorder, severe vision/language impairment, pregnancy/lactation and incomplete data.

### 2.1 Outcome measurements

Patients’ demographic and clinical data were retrieved from the EMRs. At 6 months after TBI, cognitive function was measured using the Chinese version of RBANS, which consisted of 12 tasks across five domains: immediate memory (vocabulary learning and story repetition tasks), attention (digit span and encoding tasks), language (picture naming and semantic fluency tasks), visuospatial construction (figure copy and line orientation tasks) and delayed memory functions (vocabulary recall/re-recognition, story recall and picture recall tasks). The raw scores from these tasks were standardized according to the RBANDS Stimulation Manual, and summed to generate a global cognition total score ranging from 40 to 160, with higher scores indicating better cognitive performance (Zhang et al., 2022). The threshold of 1.5 standard deviations below the age-adjusted normative RBANS mean (100 ± 15) was predefined as cognitive impairment in this study. This cutoff is a well-established, widely accepted definition of clinically significant cognitive dysfunction recommended by the Diagnostic and Statistical Manual of Mental Disorders (5th Edition) for identifying mild cognitive impairment, and has been specifically validated in multiple prior TBI cohort studies including the multi-center Transforming Research and Clinical Knowledge in TBI (TRACK-TBI) initiative, which confirmed that this threshold accurately identifies TBI patients with persistent functional cognitive deficits that require targeted clinical intervention (Hoiland et al., 2025). We pre-specified candidate predictors for inclusion in the analysis *a priori*, guided by three complementary principles: (1) previously published peer-reviewed evidence from high-quality TBI prognostic studies that demonstrated the variable’s statistically significant association with post-TBI cognitive outcomes; (2) established biological plausibility linking the variable to the pathophysiological cascades of secondary brain injury and long-term neurodegeneration after TBI; (3) consensus input from a multidisciplinary team of emergency medicine physicians, neuro-intensivists and neurorehabilitation specialists, confirming that all included variables are routinely available in acute TBI care settings to maximize the real-world clinical applicability of the final prediction model. The full pre-specified candidate predictor pool included demographic characteristics, acute radiological injury severity markers, 24-h admission laboratory test results, and post-injury 6-month clinical complication variables.

Laboratory tests including routine blood test, blood biochemical examination, serum pro-inflammatory cytokines and serum neurological function markers were measured within 24 h of admission. Neurologic consciousness was assessed by the GCS scores, which evaluated three domains including eye-opening response, verbal response and body movement. Severity was categorized into mild (GCS score between 13 and 15), moderate (GCS scores between 9 and 12) and severe (GCS score ≤8) (Zhong et al., 2025). The Acute Physiology and Chronic Health Evaluation II (APACHE II) scoring system was utilized to measure physiological condition. It was calculated based on patients’ age, temperature, mean arterial pressure, heart rate, respiratory rate, oxygenation, arterial PH value, Na^+^, K^+^, creatinine, red blood cell and white blood cell. The total scores ranged from 0 to 71, with higher scores predicting an increased risk of mortality (Zhang et al., 2024). The Sequential Organ Failure Assessment (SOFA) score was employed to estimate the status of multiple organ systems that included respiratory function, cardiovascular integrity, liver function, coagulation, renal function and neurological status. Each organ system was rated on a 5-point Likert scale from 0 (best) to 4 (worst). The total score was calculated from each component, generating a sum ranging from 0 to 24, where higher scores indicated more severe organ dysfunction (Xiang et al., 2025). Systemic Inflammation Response Index (SIRI) was calculated to assess immunoinflammatory status via the following formula: (neutrophil count × monocyte count)/lymphocyte count (Wang et al., 2025). We prospectively documented the occurrence of three important clinical complications during the hospitalization from admission to discharge: lower urinary tract symptoms (LUTS) were defined as frequent voiding, nocturia and urinary incontinence that assessed by urodynamic tests, which included measuring residual urine, urethral pressure profilometry and water cystometry (Abrams et al., 2003). Seizures were defined as observed seizure-like activity with electroencephalogram confirmation (Fordington and Manford, 2020). Post-traumatic stress disorder (PTSD) following TBI was assessed by the PTSD checklist according to the Diagnostic and Statistical Manual of Mental Disorders-5th edition (DSM-5) (Bovin et al., 2016). For the two pre-defined composite variables: (1) Elevated neurological function markers ≥2‌: the reference threshold for each individual neurological function marker was defined as the 95th percentile of measurements in the healthy local adult population, derived from the hospital’s 2023–2025 routine health checkup dataset. Patients with at least 2 out of the 4 tested markers [neuro-specific enolase (NSE), myelin basic protein (MBP), glial fibrillary acidic protein (GFAP) and S-100B] exceeding their respective 95th percentile thresholds were categorized into this group. (2) Elevated proinflammatory cytokines ≥2‌: the reference threshold for each individual proinflammatory cytokine was also set as the 95th percentile of the local healthy adult population. Patients with at least two out of the four tested markers [C-reactive protein (CRP), interleukin-6 (IL-6), interleukin-1β (IL-1β) and tumor necrosis factor-α (TNF-α)] exceeding their respective 95th percentile thresholds were categorized into this group.

### 2.2 Sample size calculation

Sample size was calculated using PASS statistical software, version 19.0 (NCSS, LLC., Kaysville, Utah, United States). Calculations were performed following two complementary, widely accepted methodological frameworks for clinical prediction model development to minimize the risk of overfitting. First, based on the published evidence, the 6-month event rate of cognitive impairment in patients with moderate–severe TBI ranges from 20 to 30% in the chronic phase (Tsai et al., 2021). We aimed to detect a minimum odds ratio of 2.0 for our key pre-specified independent variables, assuming that 1 to 50% of the cohort would present with the variable of interest. To achieve 90% statistical power with a two-sided significance level of 5%, a minimum of 385 patients was required from the perspective of traditional association testing. Second, we adhered to the minimum 10 events per variable (EPV) guideline for prediction model construction, which is the established gold standard to reduce overfitting risk in logistic regression-based prognostic models. Our pre-specified candidate predictor pool contained 9 potential variables for inclusion in the final multivariate model, requiring at least 90 observed cognitive impairment events. With the estimated 28.8% event rate of cognitive impairment in our study cohort, 385 patients would generate approximately 111 events, which meets and exceeds the 10 EPV threshold. Accounting for an estimated 20% rate of excluded or incomplete records that did not meet the study eligibility criteria, the final required sample size was determined as 482 cases.

### 2.3 Statistical analysis

Statistical analysis was performed using the SPSS software, version 22.0 (IBM Corp., Armonk, NY, United States), with a statistical significance level set at *p* < 0.05. Kolmogorov–Smirnov *Z* test was applied to assess the normality distribution. Normally distributed data, non-normally distributed data and categorical data were presented as mean ± SD, median with interquartile range (IQR) and percentage, respectively. Differences were compared using the Student *t*-test, Wilcoxon rank-sum test and chi-squared test.

All *a priori* defined candidate predictors with a univariable *p* < 0.2 were entered into the initial multivariate logistic regression model, using a forced entry variable selection method to avoid selection bias. Collinearity between included predictors was formally assessed using variance inflation factor (VIF) calculation, with VIF > 5 defined as the threshold indicating significant problematic collinearity that requires correction. Continuous variables including SIRI scores were evaluated for linearity of the logit assumption using restricted cubic spline transformation, and no significant non-linear relationship was observed, so SIRI was retained in the model as a raw continuous variable without categorization. Binary logistic regression analysis was performed to identify independent risk factors for cognitive impairment following TBI. Prognostic index (PI) was derived from the regression coefficients to distinguish patients at high risk. Receiver operating characteristic (ROC) curve was utilized to determine the optimum cutoff value, Youden’s index, specificity and sensitivity. Patients with missing data were excluded from analysis.

## 3 Results

**Figure 1:**
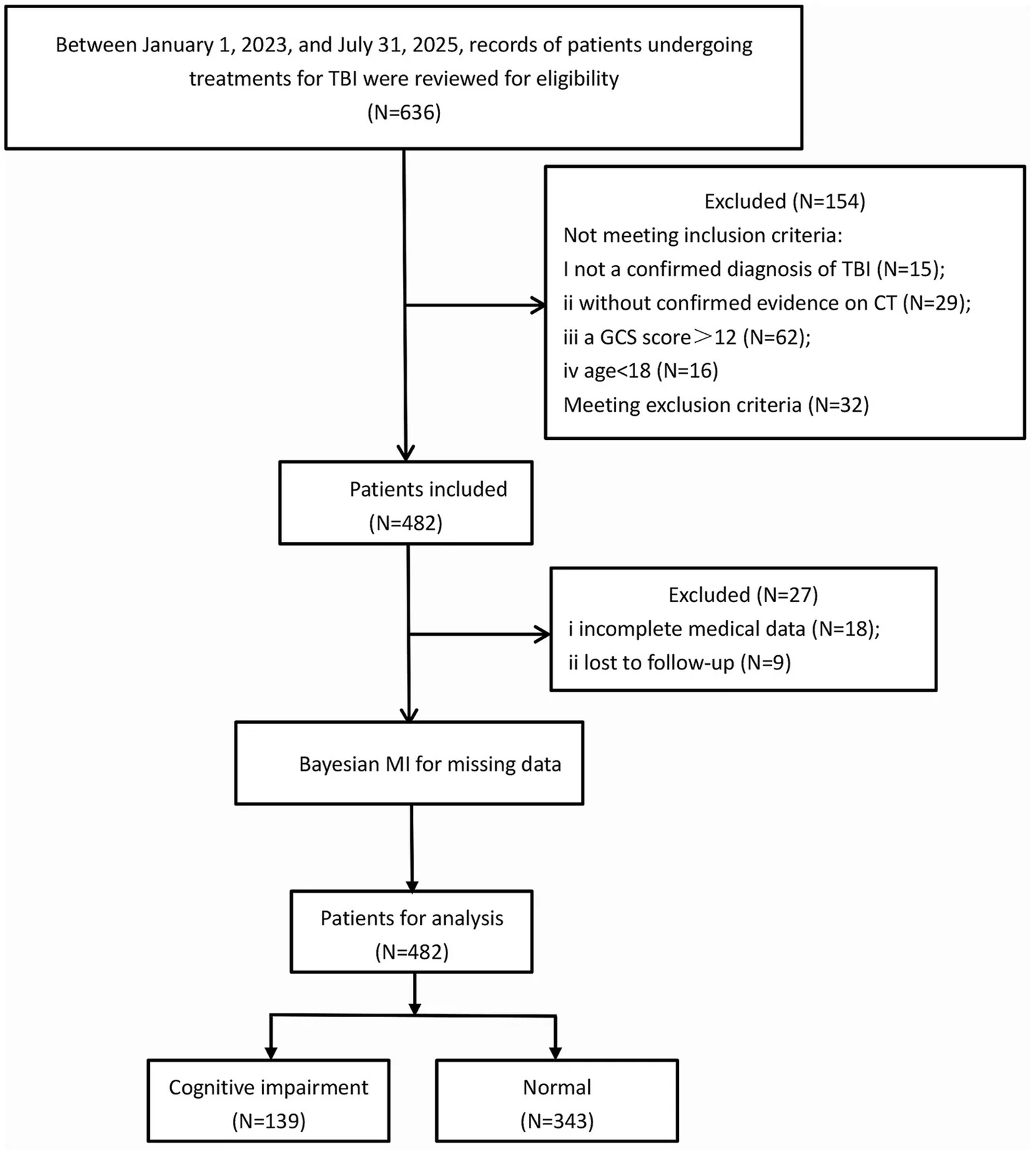
The flowchart of the study cohort. TBI, traumatic brain injury; CT, computerized tomography; GCS, Glasgow Coma Scale; MI, multiple imputation.

Figure 1 showed the flowchart of the study. The overall missing data across all variables was 5.6%, and Little’s Missing Completely at Random test confirmed the missing data pattern was non-differential (*p* = 0.372).

Demographic and clinical characteristics of the 482 included moderate–severe TBI individuals were summarized in Table 1. The overall 6-month incidence of post-TBI cognitive impairment was 28.8% (139 out of 482 patients). No statistically significant inter-group differences were observed in core baseline confounders including age, gender distribution, body mass index, TBI etiologies, baseline GCS severity stratification, total hospitalization duration and intensive care unit stay days, confirming the comparability between individuals with normal cognitive function and those who developed cognitive impairment at 6-month follow-up. The clinically meaningful, statistically significant between-group differences that were subsequently carried forward for candidate predictor evaluation were: prior history of TBI, Marshall CT classification grades IV-VI, post-injury 6-month LUTS, PTSD, elevated peripheral inflammation indicators and elevated neurological injury biomarkers. Non-significant variables that did not show univariable association with the cognitive impairment outcome at the pre-specified *p* < 0.2 threshold were excluded from subsequent multivariate variable selection to avoid unnecessary noise and reduce overfitting risk.

**Table 1:** Demographic and clinical characteristics of patients with TBI.

Variables	Cognitive function		*t*/*z*/*χ*^2^ value	*p*-value	All patients (*n* = 482)
	Normal (*n* = 343)	Impaired (*n* = 139)			
Demographic variables					
Age, years (mean ± SD)	53.74 ± 19.52	54.38 ± 18.41	−0.331	0.740	53.92 ± 17.11
Gender, *n* (%)			0.131	0.751	
Female	228 (66.5%)	90 (64.7%)			318 (66.0%)
Male	115 (33.5%)	49 (35.3%)			164 (34.0%)
Body mass index, kg/m^2^ (mean ± SD)	23.28 ± 2.23	22.97 ± 1.85	−0.269	0.788	22.31 ± 5.19
Race, *n* (%)			0.031	0.910	
Han Chinese	249 (72.6%)	102 (73.4%)			315 (72.8%)
Minority	94 (27.4%)	37 (26.6%)			131 (27.2%)
Education years, *n* (%)	9.28 ± 2.05	9.14 ± 2.26	0.695	0.510	9.24 ± 2.17
Marital status, *n* (%)			0.058	0.907	
Single/divorced	85 (24.8%)	33 (23.7%)			118 (24.5%)
Married	258 (75.2%)	106 (76.3%)			364 (75.5%)
Employment, *n* (%)			0.047	0.917	
Independent	127 (37.0%)	50 (36.0%)			177 (36.7%)
Employment/retirement	216 (63.0%)	89 (64.0%)			305 (63.3%)
Clinical variables					
Previous TBI, *n* (%)			17.660	<0.001	
No	277 (80.8%)	87 (62.6%)			364 (75.5%)
Yes	66 (19.2%)	52 (37.4%)			118 (24.5%)
TBI cause, *n* (%)			0.452	0.978	
Vehicle accident	112 (32.7%)	43 (30.9%)			155 (32.2%)
Incidental fall	93 (27.1%)	40 (28.8%)			133 (27.6%)
Violence	72 (21.0%)	27 (19.4%)			99 (20.5%)
Sport trauma	44 (12.8%)	19 (13.7%)			63 (13.1%)
Other	22 (6.4%)	10 (7.2%)			32 (6.6%)
TBI type, *n* (%)			0.211	0.995	
Epidural hematoma	42 (12.2%)	16 (11.5%)			58 (12.0%)
Subdural hemorrhage	50 (14.6%)	21 (15.1%)			71 (14.7%)
Subarachnoid hemorrhage	123 (35.9%)	49 (35.3%)			172 (35.7%)
Intracerebral hemorrhage	76 (22.1%)	30 (21.6%)			106 (22.0%)
Intraventricular hemorrhage	52 (15.2%)	23 (16.5%)			75 (15.6%)
Marshall head CT classification, *n* (%)			29.597	<0.001	
Diffuse injury I-III	114 (33.2%)	19 (12.0%)			133 (26.5%)
Diffuse injury IV	105 (30.6%)	51 (32.3%)			156 (31.1%)
Any lesion surgically removed	73 (21.3%)	45 (28.5%)			118 (23.6%)
High- or mixed-density lesion >25 cm^3^	51 (14.9%)	43 (27.2%)			94 (18.8%)
Time to hospital admission, hours (mean ± SD)	4.75 ± 1.24	4.67 ± 1.42	0.884	0.377	4.73 ± 1.39
Pupil reactivity, *n* (%)			0.117	0.943	
None reacted	52 (15.2%)	22 (15.8%)			74 (15.4%)
One reacted	105 (30.6%)	44 (31.7%)			149 (30.9%)
Both reacted	186 (54.2%)	73 (52.5%)			259 (53.7%)
Time of consciousness loss, *n* (%)			0.653	0.884	
No	119 (34.7%)	45 (32.4%)			164 (34.0%)
<30 min	116 (33.8%)	51 (36.7%)			167 (34.6%)
30 min-24 h	63 (18.4%)	27 (19.4%)			90 (18.7%)
>24 h	45 (13.1%)	16 (11.5%)			61 (12.7%)
Vital signs (mean ± SD)					
Heart rate, bpm	88.91 ± 13.88	89.26 ± 14.43	−0.284	0.804	89.01 ± 14.21
Systolic blood pressure, mmHg	116.74 ± 14.82	115.56 ± 13.97	0.802	0.423	116.40 ± 14.68
Diastolic blood pressure, mmHg	65.91 ± 10.60	66.03 ± 11.14	−0.111	0.912	65.94 ± 10.97
Respiratory rate, times/min	18.23 ± 3.87	18.45 ± 4.22	−0.551	0.582	18.29 ± 4.15
Temperature, °C	37.10 ± 0.54	37.01 ± 0.63	1.578	0.115	37.07 ± 0.59
Laboratory tests on hospital admission (mean ± SD)					
Sodium, mmol/L	140.24 ± 3.96	140.31 ± 4.67	−0.167	0.868	140.26 ± 4.38
Chloride, mmol/L	106.59 ± 5.21	107.30 ± 5.43	−1.339	0.181	106.79 ± 5.33
White blood cells count, ×10^9^ g/L	10.78 ± 2.08	12.61 ± 2.11	−8.714	<0.001	11.31 ± 2.10
Platelet count, ×10^9^ g/L	177.21 ± 10.65	177.50 ± 10.26	−0.274	0.784	177.29 ± 10.35
Lymphocytes, 10^−3^/μl	13.59 ± 9.51	15.91 ± 9.43	−2.432	0.015	14.26 ± 9.44
Red blood cell count, ×10^9^ g/L	3.76 ± 1.01	3.81 ± 1.20	−0.466	0.642	3.77 ± 1.18
Hemoglobin, g/dL	11.58 ± 2.24	11.71 ± 2.11	−0.587	0.558	11.62 ± 2.19
Albumin, g/L	23.41 ± 4.17	24.02 ± 5.13	−1.358	0.175	23.59 ± 4.55
Urea nitrogen, mmol/L	4.83 ± 1.92	4.91 ± 1.83	−0.420	0.675	4.85 ± 1.86
Creatinine, μmol/L	68.03 ± 15.13	67.84 ± 14.42	0.127	0.899	67.98 ± 14.73
International normalized ratio	1.21 ± 0.17	1.22 ± 0.25	−0.506	0.613	1.21 ± 0.32
Prothrombin time, seconds	12.74 ± 1.52	12.91 ± 2.01	−1.009	0.313	12.79 ± 1.94
Partial thromboplastin time, seconds	27.39 ± 1.65	27.52 ± 1.96	−0.741	0.459	27.43 ± 1.73
Neurological function markers within 24 h (mean ± SD)					
Neuro-specific enolase, μg/L	8.63 ± 2.35	12.57 ± 2.40	−16.573	<0.001	9.77 ± 2.38
Myelin basic protein, μg/L	2.01 ± 1.03	2.97 ± 1.15	−8.958	<0.001	2.29 ± 1.12
Glial fibrillary acidic protein, ng/L	1.15 ± 0.96	1.67 ± 0.85	−5.563	<0.001	1.30 ± 0.90
S-100B, μg/L	0.78 ± 0.23	1.03 ± 0.37	−8.957	<0.001	0.85 ± 0.30
Proinflammatory cytokines within 24 h (mean ± SD)					
C-reactive protein, mg/L	1.34 ± 0.59	2.29 ± 1.36	−10.700	<0.001	1.61 ± 0.98
Interleukin-6, pg/ml	5.76 ± 1.13	7.58 ± 1.32	−15.240	<0.001	6.28 ± 1.27
Interleukin-1β, pg/ml	31.35 ± 5.26	32.58 ± 4.98	−2.361	0.019	31.70 ± 5.02
Tumor necrosis factor-α, pg/ml	6.85 ± 1.15	7.45 ± 1.93	−11.215	<0.001	7.02 ± 1.73
Intracranial pressure, mmHg (mean ± SD)	22.31 ± 4.76	22.09 ± 5.03	0.452	0.651	22.25 ± 4.85
Common physical comorbidities, *n* (%)					
Hyperlipemia	89 (25.9%)	35 (25.2%)	0.031	0.909	124 (25.7%)
Hypertension	54 (16.0%)	24 (17.3%)	0.120	0.785	78 (16.4%)
Diabetes	45 (13.7%)	17 (12.2%)	0.178	0.766	62 (13.2%)
Other	33 (9.6%)	12 (8.6%)	0.114	0.863	45 (9.3%)
Neurological intervention, *n* (%)			0.609	0.737	
Any neurological intervention	54 (15.7%)	25 (18.0%)			79 (16.4%)
Intracranial pressure monitoring	134 (39.1%)	56 (40.3%)			213 (44.2%)
Surgery	155 (45.2%)	58 (41.7%)			190 (39.4%)
Surgery type, *n* (%)			1.491	0.684	
None	188 (54.8%)	81 (58.3%)			269 (55.8%)
Decompressive craniectomy	66 (19.2%)	29 (20.9%)			95 (19.7%)
Intracerebral hematoma evacuation	49 (14.3%)	17 (12.2%)			66 (13.7%)
Craniotomy	40 (11.7%)	12 (8.6%)			52 (10.8%)
Surgery time, hours (mean ± SD)	2.25 ± 1.27	2.33 ± 1.30	−0.622	0.534	2.27 ± 1.29
Adverse events, *n* (%)			14.004	0.016	
None	230 (67.0%)	73 (52.6%)			303 (62.9%)
Pneumonia	39 (11.4%)	15 (10.8%)			54 (11.2%)
Lower urinary tract symptoms	38 (11.1%)	28 (20.1%)			66 (13.7%)
Seizure or seizure-like activity	36 (10.5%)	23 (16.5%)			59 (12.2%)
Treatment setting, *n* (%)			0.097	0.778	
Normal inpatient	231 (67.3%)	96 (69.1%)			327 (67.8%)
Intensive care unit	112 (32.7%)	43 (30.9%)			155 (32.2%)
Hospitalization days (mean ± SD)	28.15 ± 3.52	27.98 ± 4.03	0.460	0.646	28.10 ± 3.83
Mechanical ventilation days (mean ± SD)	11.25 ± 3.64	11.30 ± 3.75	−0.135	0.892	11.26 ± 3.70
Intensive care unit stay days (mean ± SD)	13.20 ± 4.58	13.14 ± 4.06	0.134	0.893	13.18 ± 4.33
Scoring system					
GCS scores on hospital admission, *n* (%)			0.048	0.884	
Moderate (9–12)	296 (86.3%)	121 (87.1%)			417 (86.5%)
Severe (3–8)	47 (13.7%)	18 (12.9%)			65 (13.5%)
APACHE II scores on hospital admission (mean ± SD)	21.85 ± 8.78	22.03 ± 10.02	−0.275	0.783	21.90 ± 9.86
SOFA scores on hospital admission (mean ± SD)	7.26 ± 3.17	7.31 ± 3.52	−0.152	0.879	7.27 ± 3.39
SIRI scores (mean ± SD)	6.58 ± 3.55	16.93 ± 3.71	−28.620	<0.001	9.56 ± 3.68
Time since TBI to RBANS assessments (months) (mean ± SD)	6.13 ± 1.32	6.19 ± 1.54	−0.430	0.667	6.15 ± 1.47
Post-traumatic stress disorder following TBI based on DSM-5, *n* (%)	9 (2.6%)	20 (14.4%)	24.211	<0.001	29 (6.0)

TBI, traumatic brain injury; CT, computerized tomography; GCS, the Glasgow Coma Scale; APACHE, the Acute Physiologic Assessment and Chronic Health Evaluation; SOFA, the Sequential Organ Failure Assessment; SIRI, Systemic Inflammation Response Index; DSM-5, Diagnostic and Statistical Manual of Mental Disorders—5th edition; RBANS, Assessment of Neuropsychological Status; SD, standard deviation.

All candidate variables that met the pre-defined univariable screening threshold of *p* < 0.2 were entered into the initial multivariate logistic regression model using a pre-specified manual forced entry approach, rather than data-driven stepwise selection methods to minimize spurious associations. VIF assessment confirmed no problematic collinearity between any included predictors, with all VIF values <3.2, well below the standard 5.0 threshold for collinearity concern. After removing the non-significant variable of pneumonia (*p* = 0.601) from the full adjusted model, the final stable multivariate logistic regression model identified nine independent risk factors, as detailed in Table 2: TBI history [odd ratio (OR) = 2.002 (95% confidence interval (CI): 1.080–3.710)], Marshall CT classification IV [OR = 4.072 (95%CI: 1.326–12.505)]/V [OR = 4.613 (95%CI: 1.086–19.585)]/VI [OR = 5.008 (95%CI: 1.235–18.914)], elevated neurological function markers ≥2 [OR = 2.255 (95%CI: 1.247–4.081)], elevated proinflammatory cytokines ≥2 [OR = 2.209 (95%CI: 1.218–4.006)], LUTS [OR = 4.169 (95%CI: 1.264–13.751)], seizure or seizure-like activity [OR = 5.243 (95%CI: 1.330–20.662)], PTSD following TBI [OR = 5.245 (95%CI: 2.408–11.425)] and SIRI [OR = 3.066 (95%CI: 1.437–6.539)]. Using the regression coefficients derived from the final multivariate model, a validated, point-scored nomogram was constructed for easy individual-level clinical risk calculation, with the explicit prognostic index (PI) formula as follows: PI = 0.694 × TBI history (yes = 1) + 1.404 × Marshall CT classification IV (yes = 1) + 1.529 × Marshall CT classification V (yes = 1) + 1.611 × Marshall CT classification VI (yes = 1) + 0.831 × elevated neurological function markers ≥2 (yes = 1) + 0.793 × elevated proinflammatory cytokines ≥2 (yes = 1) + 1.428 × LUTS (yes = 1) + 1.657 × seizure or seizure-like activity (yes = 1) + 1.667 × PTSD (yes = 1) + 1.120 × SIRI scores −1.375.

**Table 2:** Multiple logistic regression analysis of risks factors for cognitive impairment following TBI.

Independent variables	Regression coefficient			Adjusted odds ratio			*p*-value
	B	SE	Wald χ^2^	Exp (B)	95% CI		
					Lower	Upper	
History of TBI							
No (0)							
Yes (1)	0.694	0.315	4.859	2.002	1.080	3.710	0.028
Marshall Head CT Classification			13.824				<0.001
Diffuse injury I-III (0,0,0)							
Diffuse injury IV (1,0,0)	1.404	0.572	6.016	4.072	1.326	12.505	0.014
Any lesion surgically removed (0,1,0)	1.529	0.738	4.294	4.613	1.086	19.585	0.038
High- or mixed-density lesion (0,0,1)	1.611	0.678	5.647	5.008	1.235	18.914	0.017
Elevated neurological function markers							
None (0)							
≥ Two (1)	0.813	0.303	7.228	2.255	1.247	4.081	0.007
Elevated proinflammatory cytokines							
None (0)							
≥Two (1)	0.793	0.304	6.813	2.209	1.218	4.006	0.009
Adverse events			10.376				0.001
None (0,0,0,0)							
Pneumonia (0,1,0,0)	0.279	0.534	0.273	1.322	0.464	3.769	0.601
Lower urinary tract symptoms (0,0,1,0)	1.428	0.609	5.499	4.169	1.264	13.751	0.019
Seizure or seizure-like activity (0,0,0,1)	1.657	0.700	5.606	5.243	1.330	20.662	0.018
PTSD following TBI							
No (0)							
Yes (1)	1.667	0.397	17.412	5.245	2.408	11.425	<0.001
SIRI scores	1.120	0.387	8.401	3.066	1.437	6.539	0.004
Constant	−1.375	1.637	0.706	0.253			0.401

TBI, traumatic brain injury; CT, computerized tomography; PTSD, post-traumatic stress disorder; SIRI, Systemic Inflammation Response Index; SE, standard error; CI, confidence interval.

**Figure 2:**
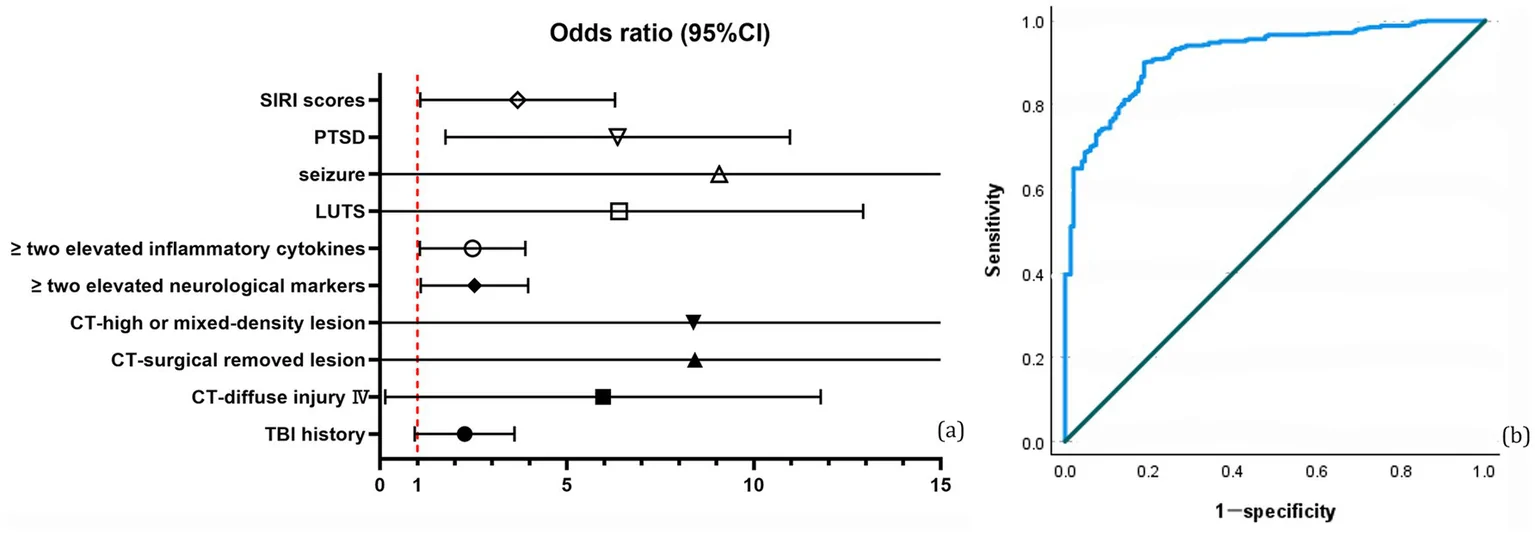
Forest plot and ROC curves for the nomogram model. **(a)** The forest plot summarized the multivariate regression of risk factors associated with cognitive function following moderate-to-severe TBI. **(b)** The AUC was 0.920 (95% CI: 0.899–0.942), demonstrating good discrimination. The Youden index value was 0.767, with a sensitivity of 90.1% and specificity of 81.2%. TBI, traumatic brain injury; CT, computerized tomography; LUTS, lower urinary tract symptoms; PTSD, post-traumatic stress disorder; SIRI, Systemic Inflammation Response Index; ROC, Receiver operating characteristic; AUC, area under the curve.

As shown in Figure 2, the area under the curve (AUC) value for the ROC curve was 0.920 (95%CI: 0.899–0.942), indicating that the prediction model demonstrated good discrimination. The Youden index was 0.767, with a sensitivity of 90.1% and specificity of 81.2%, respectively. Individuals with a PI ≥6.425 were categorized as high-risk to develop cognitive impairment after TBI, whereas those with a PI <6.425 were classified as low-risk. Kaplan–Meier analysis demonstrated a significant difference in 6-month cognitive impairment incidence between the two risk strata, with the high-risk group exhibiting a 72.3% event rate compared to only 8.7% in the low-risk group (log-rank *p* < 0.001). The non-significant Hosmer-Lemeshow goodness-of-fit test (*χ*^2^ = 7.82, df = 8, *p* = 0.451) confirmed no statistically significant deviation between predicted and observed outcomes. The overall Brier score for the full prediction model was 0.092, far below the 0.2 threshold that defines clinically useful predictive models, indicating excellent overall predictive performance.

## 4 Discussion

The present study developed and internally validated a novel, comprehensive prognostic nomogram that integrated nine routinely accessible clinical, radiological and laboratory indicators to accurately stratify the 6-month risk of cognitive impairment in adults following moderate–severe TBI. The model demonstrated excellent discriminative capacity, directly addressing a long-standing critical gap in TBI prognostic research and facilitating early prediction and supported clinicians in initiating preventative interventions for cognitive impairment at early stage.

A series of complex metabolic changes, such as reduced glucose metabolism, enhanced lipid peroxidation, disordered neurotransmitter secretion and imbalanced trace element synthesis triggered cognitive decline after brain injury (Lai et al., 2022). A recent prospective study examined the rate of long-term cognitive decline in individuals with TBI, and reported that the adjusted average decline in cognition per decade was more than twice as fast among individuals who experienced ≥2 incident TBIs (𝛽 = −0.158, *p* < 0.001) (Schneider et al., 2024). Consistent with previous findings, our results showed that adults with a history of TBI had a 2.002-fold increased risk of developing cognitive impairment after a second TBI compared to those without a prior TBI.

The Altered Cognition Scale scores, which reflected cognitive function, were significantly higher for all severities of TBI including mild (53.9 ± 21.9), moderate (54.8 ± 24.4) and severe (59.7 ± 20.9) (all *p* < 0.001). Furthermore, correlation analysis indicated that more severe TBI was associated with greater patient-reported symptoms of cognitive impairment (Wright et al., 2024). According to an earlier study involving 4,895 individuals between 1989 and 2014, the adapted Marshall CT classification categories were an objective indicator of injury severity in the acute phase of TBI (Brown et al., 2019). As a result, our findings showed that adults with higher Marshall CT classification scores (levels IV to VI) exhibited a 4.072, 4.613 and 5.008 times more likely to experience post-TBI cognitive impairment, respectively.

In individuals with TBI, a large amount of neurological function factors was released as a result of the damaged brain tissues. The protein biomarkers of NSE, MBP, GFAP, S-100B were associated with injuries to the neuronal body, white matter and astrocytes (Nishimura et al., 2022). Slavoaca et al. (2020) assessed the correlation between these protein biomarkers and neurocognitive status following TBI, which was measured by a series of neurocognitive tests including the Early Rehabilitation Barthel Index (ERBI), Glasgow Outcome Scale-Extended (GOSE), Mini-Mental State Examination (MMSE), Processing Speed Index (PSI), and Stroop Test. Their results demonstrated that the biomarker levels measured within 72 h post-injury were significant predictors of the cognitive function at 3 months (*p* < 0.05). Beyond that, TBI triggered a rapid neuroinflammatory process as early as 24 h after the injury, therefore, the activated microglia/astrocytes released inflammatory cytokines, such as CRP, IL-6, IL-1β and TNF-α (Shitaka et al., 2011). When proinflammatory dominance persisted without sufficient resolving signals, it could lead to chronic oxidative stress and progressive neurodegeneration, which contributed to the long-term cognitive impairment (Zhu et al., 2026). In addition, the composite SIRI, which was derived from hematological neutrophils, monocytes and lymphocytes could accurately reflect the complex interaction between inflammation and immune status, providing a significant insight into the peripheral inflammation. This inflammation disrupted the blood–brain barrier and subsequently triggered neuroinflammation, leading to worsened neurodegeneration and cognitive dysfunction. Hence, the elevated level of SIRI was linked to a lower odd of normal cognitive performance (OR = 0.891, *p* < 0.001) (Wang et al., 2025). In line with earlier evidence, our results showed that adults with two or more elevated neurological function markers or proinflammatory cytokines were 2.255 and 2.209 times more likely, respectively, to experience cognitive impairment at 6 months after a TBI. Additionally, higher levels of SIRI (OR = 3.066) were significantly associated with an increased risk of cognitive impairment following TBI.

In addition to these findings, patients who experienced LUTS, seizures, or PTSD within 6 months following a TBI in the present study had a 4.169, 5.243, and 5.245 times higher risk of developing cognitive impairment, respectively. Although the micturition center was located in the frontal lobe, diffuse axonal injuries (DAI) after TBI usually contributed to the urologic dysfunction with LUTS (Giannantoni et al., 2011). However, the grade of DAI was found to be negatively correlated with cognitive function (*r* = −0.403, *p* < 0.001), supporting our results (Chen et al., 2023). Post-traumatic seizure was a common consequence in individuals with moderate or severe TBI. Based on a recent multicenter cohort study, seizure was linked to a 40% increased risk of cognitive impairment, and when both TBI and seizure were present, the risk increased by 57% in these individuals (Zhu et al., 2024). Regarding PTSD, a prior study investigated neurocognitive problems in 1,134 civilians who suffered probable PTSD following TBI, and found that PTSD was significantly associated with poorer cognitive test performance, including higher scores on the Trail Making Test (TMT) (OR = 1.35) and lower scores on the Rey Auditory Verbal Learning Test (RAVLT) (OR = 0.74) (both *p* < 0.001) (Van Praag et al. 2022).

The primary clinical value of our nomogram lied in its practicality and potential for seamless integration into routine acute TBI care workflows. All nine included variables were either standard demographic information, routinely collected clinical data, or widely available laboratory tests in most tertiary care centers, requiring no specialized equipment or additional patient burden. This suggested that implementing our model could help optimize the allocation of limited cognitive rehabilitation resources by accurately identifying the high-risk patient subgroup that would derive the greatest benefit from early, intensive intervention, while safely deferring routine follow-up for low-risk patients, thereby improving healthcare efficiency and patient outcomes.

This study had several limitations. First, the retrospective single-center design introduces potential selection bias and limits the generalizability of our findings to other patient populations and healthcare settings. Second, several exclusion criteria might introduce selection bias, particularly excluding non-survivors, patients with incomplete records and those lost to follow-up. Consequently, the derived prediction model could not fully represent the broader spectrum of all admitted patients. Third, several important factors known to influence cognitive outcomes after traumatic brain injury were not considered. These included the potential effects of medications, particularly antiseizure medications and other psychotropic agents, time since TBI, premorbid cognitive or functional status, and the level of functional disability after injury. The omission of these potentially important confounders might have influenced the observed associations and should be recognized when interpreting the findings. Future studies should incorporate these variables to improve the robustness and clinical applicability of the prediction model. Four, without baseline cognition, it was difficult to determine whether impairment observed at follow-up was entirely attributable to TBI. Five, since the study lacked both internal and external validity, prospective multi-center cohorts were essential to confirm its generalizability and transportability before widespread clinical implementation. Last, the cognitive impairment outcome was assessed at a single 6-month timepoint; future studies with longer follow-up durations and repeated cognitive assessments were needed to evaluate the model’s predictive accuracy for long-term cognitive trajectories. Future studies should involve a well-designed randomized controlled trial to confirm our results.

## 5 Conclusion

In conclusion, this study developed a clinically practical nomogram model for predicting 6-month cognitive impairment in adults with moderate-to-severe TBI. Despite some limitations, these findings might assist healthcare providers in optimizing the early management of cognitive impairment after TBI and improving patient outcomes in a clinical setting.

## Data Availability

The raw data supporting the conclusions of this article will be made available by the authors, without undue reservation.

## Abbreviations

TBI: traumatic brain injury
RBANS: Repeatable Battery for the Assessment of Neuropsychological Status
EMRs: electronic medical records
CT: computerized tomography
GCS: Glasgow Coma Scale
CRP: C-reactive protein
IL-6: interleukin-6
IL-1β: interleukin-1β
TNF-α: tumor necrosis factor-α
NSE: neuro-specific enolase
MBP: myelin basic protein
GFAP: glial fibrillary acidic protein
APACHE II: Acute Physiology and Chronic Health Evaluation II
SOFA: Sequential Organ Failure Assessment
SIRI: Systemic Inflammation Response Index
LUTS: lower urinary tract symptoms
PTSD: post-traumatic stress disorder
SD: standard deviation
IQR: inter quartile range
PI: prognostic index
ROC: receiver operating characteristic
MI: multiple imputation
OR: odds ratio
CI: confidence interval
